# Quality of life of people with and without respiratory complications in post-COVID-19 syndrome: a retrospective study

**DOI:** 10.1590/0034-7167-2025-0195

**Published:** 2026-08-21

**Authors:** Larissa Bento de Araújo Mendonça, Francisca Elisangela Teixeira Lima, Lívia Maia Pascoal, Paulo César de Almeida, Patricia Neyva da Costa Pinheiro, Glaubervania Alves Lima, Maria Gabriela Miranda Fontenele, Lorena Pinheiro Barbosa

**Affiliations:** I Universidade Federal do Ceará. Fortaleza, Ceará, Brazil; II Universidade Federal do Maranhão. Imperatriz, Maranhão, Brazil; III Universidade Estadual do Ceará. Fortaleza, Ceará, Brazil

**Keywords:** Quality of Life, Post-Acute COVID-19 Syndrome, Retrospective Studies, Respiratory Tract Infections, Nursing., Calidad de Vida, Síndrome Post Agudo de COVID-19, Estudios Retrospectivos, Infecciones del Sistema Respiratorio, Enfermería.

## Abstract

**Objectives:**

to assess and compare the quality of life (QoL) of people with and without respiratory complications resulting from post-COVID-19 syndrome.

**Methods:**

a retrospective, comparative, and analytical observational study, with a quantitative approach and non-probabilistic convenience sampling, conducted with 300 people residing in Ceará, Maranhão, or Pernambuco, distributed into case group (n=100/with respiratory complications) and control group (n=200/without respiratory complications). For data collection (2021-2022), the Medical Outcomes Study 36-Item Short-Form Health Survey (SF-36) and the St George’s Respiratory Questionnaire (SGRQ) were used. Student’s t-test, ANOVA, and Pearson correlation were performed (p<0.05).

**Results:**

the case group presented lower scores in all SF-36 domains (p<0.05), indicating worse QoL. In the SGRQ, symptoms, activity, and impacts showed greater impairment (p<0.001). An inverse linear correlation was observed between SF-36 and SGRQ scores.

**Conclusions:**

people with post-COVID-19 respiratory complications presented worse QoL, highlighting the relevance of multidisciplinary follow-up in respiratory rehabilitation for improving QoL.

## RESUMEN

**Objetivos:**

evaluar y comparar la calidad de vida (CV) de personas con y sin complicaciones respiratorias derivadas del síndrome post-COVID-19.

**Métodos:**

estudio observacional retrospectivo, comparativo y analítico, con enfoque cuantitativo y muestreo no probabilístico por conveniencia, realizado con 300 personas residentes en Ceará, Maranhão o Pernambuco, distribuidas en un grupo de casos (n=100/con complicaciones respiratorias) y un grupo control (n=200/sin complicaciones respiratorias). Para la recolección de datos (2021-2022), se utilizaron la *Medical Outcomes Study 36-Item Short-Form Health Survey* (SF-36) y el *St George’s Respiratory Questionnaire* (SGRQ). Se realizaron pruebas t de Student, ANOVA y correlación de Pearson (p<0,05).

**Resultados:**

el grupo de casos presentó puntuaciones más bajas en todos los dominios del SF-36 (p<0,05), lo que indica una peor CV. En el SGRQ, los síntomas, la actividad y los impactos psicosociales mostraron un mayor deterioro (p<0,001). Se observó una correlación lineal inversa entre las puntuaciones del SF-36 y del SGRQ.

**Conclusiones:**

las personas con complicaciones respiratorias post-COVID-19 presentaron una peor CV, lo que resalta la importancia del seguimiento multidisciplinario en rehabilitación respiratoria para mejorar la CV.

## INTRODUCTION

Respiratory dysfunction is among the most prevalent complications in people who have developed post-COVID-19 syndrome, a condition characterized by the persistence of signs and symptoms of the disease for more than four weeks after acute infection. These symptoms include cough, fatigue, dyspnea, headache, neurocognitive difficulties, mental changes, anosmia, dysgeusia, among others, negatively affecting the quality of life of those affected^(1-6)^.

Among the persistent symptoms most associated with the syndrome, dyspnea stands out. In the United States, a study identified this symptom as one of the most frequently reported by participants with post-COVID-19 syndrome, associating it with impaired quality of life and increased dependence on others for self-care activities^(7)^. In Brazil, considering a prevalence of 10%, it is estimated that approximately 4 million Brazilians have developed this syndrome^(8-10)^.

Many patients who developed post-COVID-19 syndrome reported persistent symptoms that limited their ability to work and participate socially. Previously healthy individuals began experiencing unexpected symptoms, while those with pre-existing comorbidities faced a worsening of their clinical conditions, contributing to impaired functionality and quality of life^(11,12)^.

Quality of life, as a multidimensional concept, refers to an individual’s perception of their position in life, considering the cultural context and value system in which they are embedded, as well as their goals, expectations, standards, and concerns. Furthermore, it is directly related to various factors of human life, such as health, financial situation, work, family, environment, leisure, and others^(13)^.

In this context, assessing the quality of life of people with post-COVID-19 syndrome is essential to understanding the impact of respiratory changes resulting from the infection. To this end, different instruments have been proposed in the literature to assess this outcome, both in general populations and in groups with specific diseases^(14)^.

Given the global impacts of COVID-19 on the health and functionality of populations, it is essential to study and understand post-COVID-19 syndrome in its clinical and social dimensions, as well as to predict its cumulative impact, monitor hospitalization and mortality rates, and estimate its prevalence^(15,16)^. Although global literature more broadly addresses post-COVID-19 sequels, there is still a gap in studies investigating quality of life and respiratory complications in different regional contexts in Brazil, especially in the Northeast, where socioeconomic particularities and access to healthcare services can influence these outcomes.

Thus, gathering data on the quality of life of people who developed respiratory complications resulting from post-COVID-19 syndrome will provide information that can support the planning of disease prevention and health promotion programs, considering micro and macro-social influences in the context of health actions^(17)^. It is hypothesized that people with respiratory complications resulting from post-COVID-19 syndrome have a worse quality of life compared to those without complications.

### Study relevance

The relevance of this study lies in highlighting the impact of post-COVID-19 respiratory conditions on the quality of life of people residing in northeastern Brazil, a region marked by social inequalities and historical challenges in accessing healthcare services. Post-COVID-19 syndrome has generated increasing demand for healthcare, especially among people who develop persistent respiratory complications. However, regional investigations that comparatively assess the impact of these complications on quality of life are still scarce, particularly in northeastern Brazil, where socioeconomic inequalities and limited access to services can intensify this impairment. The results of this study offer evidence that can guide health surveillance actions, respiratory rehabilitation practices, and multidisciplinary follow-up strategies, contributing to the planning of public policies and the improvement of care for the affected population.

This article is the product of a thesis^(18)^ deposited in an institutional repository: http://repositorio.ufc.br/handle/riufc/70986.

## OBJECTIVES

To assess and compare the quality of life of people with and without respiratory complications resulting from post-COVID-19 syndrome.

## METHODS

### Ethical aspects

The study was approved by the Research Ethics Committee, in accordance with the ethical precepts of Resolution 466/2012 of the Brazilian National Health Council, under Opinion 4,278,495/2020 and Certificate of Presentation of Ethical Appraisal 36193820.1.0000.5054.

### Study design

This is a retrospective observational study, comparative and analytical in nature, with a quantitative approach, developed in three states of northeastern Brazil: Ceará, Maranhão, and Pernambuco. The recommendations for observational studies were followed according to Strengthening the Reporting of Observational studies in Epidemiology^(19)^.

### Population, sample, and selection criteria

The study population consisted of individuals diagnosed with COVID-19, whose data were initially identified in the Influenza Epidemiological Surveillance Information System (In Portuguese, *Sistema de Informação da Vigilância Epidemiológica da Gripe* - SIVEP-*GRIPE*) and the e-SUS *Notifica* system. From this population, the selected sample was organized into two groups: a case group and a control group. The case group consisted of individuals over 18 years of age who experienced respiratory complications during the illness, persisting for at least four weeks after COVID-19 diagnosis, characterizing post-COVID-19 syndrome, and who became ill between March 2020 and January 2022. The control group consisted of individuals who met the same criteria as the case group, except for having experienced respiratory complications resulting from post-COVID-19 syndrome.

Participants who did not fully complete the questionnaires, who completed them after the data collection period ended, or who lacked the cognitive capacity to answer the questionnaires were excluded, totaling 27 exclusions. Missing data handling was performed using a listwise exclusion method, retaining only the fully valid questionnaires in the final analysis.

Sample size calculation was based on the formula for case-control studies (n= z^2^_5%_ {1/[ P_1_(1 - P_1_)] + 1/[P_2_(1 - P_2_)]} / [log_e_(1- ɛ)]^2^), considering an odds ratio of 2, a probability of 10% of patients with chronic obstructive pulmonary disease, a relative accuracy of 50%, and a significance level of 10%, resulting in 100.

In order to provide a more accurate estimate of the frequency of exposure in the control group and to increase the study’s power, thus increasing the ability to detect an association, if it is indeed present, it was decided to use twice the sample size calculated for the control group, totaling 200 people.

### Study protocol

Firstly, data were collected from SIVEP-*GRIPE* and e-SUS *Notifica*, which contain the notification form for cases of patients with COVID-19 and compiled by the Epidemiological Surveillance of the State Health Departments (In Portuguese, *Secretaria Estadual de Saúde* - SESA) of the states of Ceará, Maranhão and Pernambuco. Access authorization and data use were obtained through a formal request to the respective SESAs. Subsequently, a link was sent via instant messaging application for participants to access and complete a form if they agreed to participate in the research. In both groups, a validated form for characterizing people who had COVID-19^(20)^ was applied, containing questions about research participant sociodemographic and clinical characteristics, and the Medical Outcomes Study 36-Item Short-Form Health Survey (SF-36), validated for use in Brazil^(14)^. The Saint George’s Respiratory Questionnaire (SGRQ) was applied only to those who reported having respiratory complications, with the aim of assessing the impact on the quality of life of this group.

The SF-36 quality of life questionnaire assesses participants’ quality of life over the past four weeks. It consists of 36 items, divided into eight domains: 1 - Physical functioning (question 3, with ten items); 2 - Role limitations due to physical health problems (question 4, with four items); 3 - Bodily pain (questions 7, with one item, and 8, with one item); 4 - General health perceptions (questions 1, with one item, and 11, with four items); 5 - Energy/fatigue (question 9, with four items corresponding to the letters: a + e + g + i); 6 - Social functioning (questions 6, with one item, and 10, with one item); 7 - Role limitations due to personal or emotional problems (question 5, with three items); 8 - Emotional well-being (question 9, with five items represented by the letters: b + c + d + f + h); There is one question (question 2) that assesses changes in health status, but it is not used in the calculation of the domains, being applied only to assess how much better or worse an individual’s health is compared to a year ago^(21)^.

To assess the results, after application, a score is given for each question, which is subsequently transformed into a scale from zero to 100, with zero being the worst health status and 100 the best. Each dimension is analyzed separately^(14)^.

The SGRQ, used in the case group, is composed of three domains: symptoms; activity; and impacts. The value for the symptoms domain is calculated by summing all the scores from the questions in Part 1 of the questionnaire, where each question’s score can range from zero to 93.3, and dividing the value by 662.5. The activity domain is calculated by adding all the scores from sections 2 and 6 of part 2 of the questionnaire, where each question’s score can range from zero to 90.6, and dividing the value by 1.209.1. The impacts domain is calculated by adding all the scores from sections 1, 3, 4, 5, and 7 of questionnaire 2, where each question’s score can range from zero to 96.7, and dividing the value by 2.117.8. The total is referred to as a maximum percentage. Values close to zero represent a better quality of life in that domain^(22)^.

It is important to note that the SGRQ was only applied to the case group, as it is a specific instrument for assessing the quality of life of people with chronic respiratory symptoms. The analyses involving the SGRQ were limited to describing the scores within the case group and analyzing the correlation with the SF-36 domains, which was also applied to this group.

Data collection took place between November 2021 and April 2022. Initially, data were collected from COVID-19 case notification forms entered and available in the notification systems of the Brazilian Ministry of Health (SIVEP-*GRIPE* and e-SUS *Notifica*) from the states of Ceará, Maranhão, and Pernambuco. Following prior authorization from the SESA of each state, data were exported to the COVID-19 and respiratory syndrome monitoring software, developed and validated in Brazil^(20)^.

Afterwards, the software generated a link, which was sent by the researchers themselves to previously registered individuals with COVID-19 via a messaging app. The link included the following question: have you had COVID-19 and do you reside in the states of Ceará, Maranhão, or Pernambuco? Participants who answered positively to the question, upon clicking the link, gained access to the Informed Consent Form. After agreeing to participate in the research, they were automatically directed to complete the characterization form and the SF-36. Participants who reported developing respiratory complications resulting from COVID-19 were allocated to the case group and, in addition to the instruments mentioned above, were also directed to complete the SGRQ.

Data reliability was ensured by linking the electronic form to the CPF (Brazilian taxpayer ID), telephone number, and email address previously registered in the official notification systems (SIVEP-*GRIPE* and e-SUS *Notifica*), which prevented duplicate responses and allowed verification of participants’ identity.

### Analysis of results and statistics

Data were processed and analyzed using the Statistical Package for the Social Sciences version 20.0, under license 10101131007. Variable normality was verified using the Kolmogorov-Smirnov test. Means were compared using the parametric Student’s t-test and ANOVA. Associations among categorical variables were analyzed using the chi-square test or Fisher’s exact test. Inferential analyses with p<0.05 were considered statistically significant.

## RESULTS

Sociodemographic, clinical, and lifestyle characteristics were assessed for homogeneity between the groups and showed no significant differences (p>0.05), meaning that both groups were similar to each other, as shown in Table 1.

**Table 1:** Distribution of people with COVID-19 according to sociodemographic, clinical and lifestyle characteristics in the case group and control group (N=300), Fortaleza, Ceará, Brazil, 2022

Variables	Case group		Control group		*p* value^*^
	N=100	%	N=200	%	
Age					0.410
<60	95	95	187	93.5	
>60	5	5	13	6.5	
**Sex**					0.557
Female	64	64	121	60.5	
Male	36	36	79	39.5	
**Education**					0.828
Up to high school	19	19	45	22.5	
Higher education	71	71	134	67	
Graduate education	10	10	21	10.5	
**Profession**					0.508
Healthcare professional	24	24	47	23.5	
Professional from other fields	50	50	112	56	
Other	26	26	41	20.5	
**Marital status**					0.190
Single	42	42	87	43.5	
Married/common-law marriage	52	52	88	44	
Divorced/separated/widowed	6	6	25	12.5	
**State of residence**					0.810
Ceará	71	71	128	64	
Maranhão	27	27	47	23.5	
Pernambuco	2	2	25	12.5	
**Clinical conditions prior to COVID-19**					
Respiratory allergies	23	23	36	18	0.304
Diabetes	5	5	15	7.5	0.413
Lung disease	4	4	0	0	0.098
High blood pressure	16	16	23	11.5	0.275
Obesity	10	10	12	6	0.210
Overweight	16	16	32	16	1.000
None	41	41	105	52.5	
**COVID-19 symptoms**					
Adynamia/fatigue	24	24	20	10	0.352
Anosmia	26	26	76	38	0.052
Headache	46	46	91	45.5	0.935
Runny nose/phlegm	22	22	44	22	1.000
Dysgeusia	27	27	67	33.5	0.253
Dyspnea	18	18	22	11	0.490
Sore throat	30	30	51	25.5	0.408
Body aches	49	49	55	27.5	0.134
Fever	46	46	96	48	0.744
Cough	29	29	56	28	0.856
Other	37	37	53	26.5	0.059
No symptoms	1	1	15	7.5	
**Developed complications in post-COVID-19 syndrome (except respiratory)**					0.377
Yes	75	75	87	43.5	
No	25	25	113	56.5	
**Types of complications developed**					
Adynamia/fatigue	31	31	15	7.5	0.587
Anosmia	7	7	10	5	0.480
Anxiety	11	11	7	3.5	0.085
Headache	8	8	9	4.5	0.216
Concentration deficit	5	5	8	4	0.388
Memory deficit	10	10	19	9.5	0.890
Insomnia	6	6	3	1.5	0.069
Hair loss	22	22	21	10.5	0.274
**Physical activity**					0.412
Yes	51	51	161	80.5	
No	49	49	39	19.5	
**Smoking**					1.00
Yes	4	4	9	4.5	
No	96	96	191	95.5	
**Alcoholism**					0.406
Yes	44	44	78	39	
No	56	56	122	61	

* *Teste qui-quadrado ou teste exato de Fisher.*

Most participants, from both groups (case and control), were under 60 years of age (mean 35.95 ± 12.1 years), were female, had higher education (complete or incomplete), were not healthcare workers, were married/in a stable relationship, and resided in the state of Ceará.

The most common pre-existing clinical conditions included respiratory allergies, overweight, and hypertension (HT), while body aches, fever, and headache were the most frequently reported COVID-19 symptoms. Adynamia/fatigue, hair loss, and anxiety were the most prominent manifestations of post-COVID-19 syndrome, with persistent cough, dyspnea, and fatigue on exertion being the prevalent respiratory complications in the group. Pre-COVID-19 lifestyle was characterized by regular physical activity and low rates of smoking and alcohol consumption.

In the SF-36 quality of life assessment, participants with respiratory complications (case group) showed significantly lower means in all eight domains compared to patients without these complications (control group), indicating a worse quality of life in the case group. The differences found were clinically and statistically significant in all domains, as shown in Table 2. It is noteworthy that the greatest negative impacts were identified in the bodily pain, role limitations due to physical health problems, and physical functioning domains.

**Table 2:** Comparison of mean domain scores from the Medical Outcomes Study 36-Item Short-Form Health Survey, standard deviation, and p-value in the case and control groups (N=300), Fortaleza, Ceará, Brazil, 2022

Domains	Group	n	Mean (+SD)	Mean difference	*p* value^*^
**Physical functioning**	Case	100	45.3(+18.2)	-7.9	**<0.001**
	Control	200	53.2(+15.9)		
**Role limitations due to physical health problems**	Case	100	29.3(+21.1)	-9.8	**<0.001**
	Control	200	39.1(+17.2)		
**Bodily pain**	Case	100	51.7(+19.7)	-11.3	**<0.001**
	Control	200	63.0(+18.6)		
**General health perceptions**	Case	100	47.8(+15.9)	-6.2	**<0.001**
	Control	200	54.0(+14.3)		
**Energy/fatigue**	Case	100	39.6(+18.1)	-6.9	**<0.001**
	Control	200	46.5(+17.3)		
**Social functioning**	Case	100	48.8(+22.0)	-6.6	**0.010**
	Control	200	55.4(+20.2)		
**Role limitations due to personal or emotional problems**	Case	100	29.0(+22.0)	-6.0	**0.021**
	Control	200	35.0(+20.0)		
**Emotional well-being**	Case	100	45.4(+17.0)	-7.8	**<0.001**
	Control	200	53.2(+15.9)		

* *Teste t de Student; DP - desvio padrão.*

In the case group, the SGRQ domain assessment revealed that the symptoms domain presented scores ranging from zero to 88.1; the activity domain ranged from zero to 100; and the impacts domain ranged from zero to 99.3. The mean score for the symptoms domain (33.2) was the lowest compared to the impacts (41.2) and activity (57.6) domains, with a statistically significant difference in all domains (p<0.001) (Table 3), indicating a better quality of life in the symptoms domain and a worse one in the activity domain.

**Table 3:** Analysis of means, standard deviations, and p-values of the St. George’s Respiratory Questionnaire domains in the case group (N=100), Fortaleza, Ceará, Brazil, 2022

SGRQ domains (N=100)	Mean (+SD)	*p* value^*^
Symptoms	33.2(+23.1)	**<0.001**
Activity	57.6(+34.3)	**<0.001**
Impacts	41.2(+27.4)	**<0.001**

SD - standard deviation; SGRQ - St George’s Respiratory Questionnaire;

* ANOVA p<0.001.

In the linear correlation analysis (Table 4), it was observed that all SGRQ domains were directly proportional to each other (p<0.001). Similarly, the eight SF-36 domains were directly proportional to each other (p<0.001).

**Table 4:** Linear correlation analysis between the Medical Outcomes Study 36-Item Short-Form Health Survey and the St. George’s Respiratory Questionnaire domains in the case and control groups (N=300), Fortaleza, Ceará, Brazil, 2022

		SGRQ domains		SF-36 domains							
		Activity	Impacts	Physical functioning	Role limitations due to physical health problems	Bodily pain	General health perceptions	Energy/fatigue	Social functioning	Role limitations due to personal or emotional problems	Emotional well-being
**SGRQ domains**											
Symptoms	r	0.679	0.732	-0.207	-0.219	-0.148	-0.211	-0.109	-0.195	-0.095	-0.178
	p	**<0.001**	**<0.001**	**0.039**	**0.028**	0.141	**0.035**	0.280	0.052	0.346	0.077
Activity	r		0.836	-0.368	-0.514	-0.428	-0.330	-0.398	-0.346	-0.277	-0.341
	p		**<0.001**	**<0.001**	**<0.001**	**<0.001**	**0.001**	**<0.001**	**<0.001**	**0.005**	**0.001**
Impacts	r			-0.355	-0.514	-0.363	-0.325	-0.361	-0.424	-0.361	-0.350
	p			**<0.001**	**<0.001**	**<0.001**	**0.001**	**<0.001**	**<0.001**	**<0.001**	**<0.001**
**SF-36 domains**											
Physical functioning	r				0.500	0.513	0.323	0.488	0.521	0.296	0.406
	p				**<0.001**	**<0.001**	**<0.001**	**<0.001**	**<0.001**	**<0.001**	**<0.001**
Role limitations due to physical health problems	r					0.539	0.288	0.476	0.501	0.529	0.372
	p					**<0.001**	**<0.001**	**<0.001**	**<0.001**	**<0.001**	**<0.001**
Bodily pain	r						0.414	0.483	0.520	0.341	0.376
	p						**<0.001**	**<0.001**	**<0.001**	**<0.001**	**<0.001**
General health perceptions	r							0.531	0.436	0.330	0.581
	p							**<0.001**	**<0.001**	**<0.001**	**<0.001**
Energy/fatigue	r								0.673	0.505	0.759
	p								**<0.001**	**<0.001**	**<0.001**
Social functioning	r									0.561	0.646
	p									**<0.001**	**<0.001**
Role limitations due to personal or emotional problems	r										0.497
	p										**<0.001**

*r - Pearson’s correlation coefficient; p - significance value of the test; SGRQ - St George’s Respiratory Questionnaire; SF-36 - Medical Outcomes Study 36-Item Short-Form Health Survey.*

Regarding the correlation between the SGRQ and SF-36 domains, the SGRQ symptoms domain was inversely proportional to three SF-36 domains: physical functioning (p=0.039); role limitations due to physical health problems (p=0.028); and general health perceptions (p=0.035). Meanwhile, the activity and impacts SGRQ domains were inversely proportional to all eight SF-36 domains (p<0.0001).

These findings demonstrate that the scales showed a statistically significant inversely proportional correlation, indicating that the higher the mean scores achieved in the SF-36 domains, the lower the scores achieved in the SGRQ domains. This shows that the questionnaire domains correlate with each other and present similar interpretations in terms of total score analysis. For the SF-36, the higher the score obtained in that domain, the better the individual’s quality of life, while in the SGRQ, the quality of life in that domain improves as the scores decrease.

## DISCUSSION

This study investigated the impact of post-COVID-19 syndrome on the quality of life of individuals residing in northeastern Brazil, focusing on respiratory complications.

The regional nature of this study, conducted in three states in northeastern Brazil, broadens the representativeness of the findings and contributes to the national and Latin American literature by highlighting sociocultural particularities and challenges in accessing healthcare services that influence the quality of life of people affected by post-COVID-19 syndrome.

Participant sociodemographic characteristics, mostly young adults, female, with higher education and professionally active, converge with profiles identified in studies carried out in Portugal and Brazil (Ribeirão Preto, São Paulo)^(23,24)^. This consistency suggests that, although the infection can affect all age groups, younger and more active populations also face significant long-term consequences, challenging the initial perception that only older adults would be severely impacted.

The comorbidities mentioned in this study support the findings of research conducted in Denmark that investigated patients diagnosed with post-COVID-19 syndrome, finding that 87.5% of them had some pre-existing comorbidity, predominantly HT, emphysema, asthma, ulcerative colitis, diabetes, and cardiovascular diseases^(16)^. Brazilian studies have also identified HT and pre-diabetes/diabetes as frequent pre-existing comorbidities^(5,25)^.

Research conducted in Brazil with 470 people affected by COVID-19 found a high prevalence of respiratory symptoms, ranking third among the most frequently reported symptoms, surpassed only by fatigue and alopecia^(26)^. Among the respiratory complications of post-COVID-19 syndrome, dyspnea is one of the most prevalent symptoms, as evidenced by studies conducted in different countries^(8,27-29)^.

It is noteworthy that the prevalence of pre-existing comorbidities and the severity of the initial infection may act as confounding factors in the assessment of post-COVID-19 quality of life. The absence of a more detailed stratification regarding the severity of the infection and the confirmation of the presence of respiratory comorbidities in case-control analysis may have attenuated the strength of the associations found.

The healthy lifestyle adopted by participants in this research was also observed in another study, reinforcing that regular physical activity, a balanced diet, abstinence from smoking and alcohol, and the proper use of medication contribute to the prevention of diseases, functional decline, longevity, and a better quality of life^(30)^.

In this study, the quality of life of people with respiratory complications resulting from post-COVID-19 syndrome (case group) was lower than that of those without such complications (control group). This result can be explained by respiratory system impairment during COVID-19, mainly due to the exacerbated immune response of the host and inflammatory organ damage in the acute phase of the disease. Pulmonary function tests demonstrated a reduction in forced vital capacity, impaired gas exchange, and reduced respiratory muscle strength, requiring respiratory rehabilitation^(31)^.

A study with 20 older adults diagnosed with COVID-19 showed scores below 60 points in all SF-36 domains, suggesting a low quality of life^(32)^. On the other hand, research conducted in Minas Gerais with 114 participants showed that the majority of participants presented scores above 50 points in all SF-36 domains. Men showed better results than women in the physical functioning, bodily pain, social functioning, and role limitations due to personal or emotional problems domains^(33)^.

In the context of healthcare professionals, a study conducted with 33 nurses working in an Intensive Care Unit of a hospital in Rio de Janeiro revealed significant physical and psychological impairment, reflected in the general health perceptions and energy/fatigue domains^(13)^. Overall, the impact of the pandemic was significant: in Bahia, a study with 2,920 workers with COVID-19 showed that 8.9% reported some disability resulting from the disease^(34)^. Another study showed that the greatest impact of COVID-19 was on work, with almost half of the participants working reduced hours, and 23.3% unable to return to work^(28)^. Furthermore, 16% of individuals with post-COVID-19 syndrome reported impairment in participating in sports or recreational activities^(35)^.

It is important to highlight that post-COVID-19 syndrome symptoms can persist for months, even in mild cases of the disease^(16)^. In the present study, according to the SGRQ, the case group showed better quality of life in the symptoms domain and worse in the activity domain. This finding is consistent with a study that also assessed quality of life using the SGRQ, finding that the group of 26 people with respiratory complications from COVID-19 had worse quality of life compared to the control group, made up of 26 healthy people^(36)^.

Different comorbidities are associated with the severity of symptoms and the negative impact on quality of life. Factors such as age, smoking, diabetes, hypothyroidism, and asthma have been linked to slower recovery and improved quality of life^(37)^. Stigma associated with COVID-19 has become widespread, potentially resulting in a sense of hopelessness. Increasing reports of prolonged malaise and exhaustion have contributed to the emergence of emotional disorders, anxiety, and psychological distress, significantly affecting quality of life^(38)^.

Given this context, quality of life assessment instruments become valuable tools for quantifying, in subjective and objective terms, the health disturbances perceived by patients and professionals. These instruments are applicable to different types of diseases, treatments, or interventions, across different cultures and locations, and their scores can be analyzed in distinct ways^(22)^. In the present study, a linear correlation was observed between the SF-36 and SGRQ domains, in which both demonstrated an inversely proportional correlation with each other, since higher scores on the SF-36 indicate better quality of life, while, on the SGRQ, lower scores are related to better conditions^(14,22)^.

Therefore, it is imperative that the care of people who have developed complications from post-COVID-19 syndrome be carried out with an interdisciplinary approach and supported by an integrated research agenda to avoid fragmentation of healthcare and allow for monitoring of the long-term consequences of post-COVID-19 syndrome^(38)^. The findings of this study have significant implications for nursing practice, especially in the context of rehabilitation and continuing care.

### Study limitations

Limitations include the possibility of self-report bias, including recall bias by research participants, since the data were collected from past information. The use of online forms may have introduced selection bias, limiting participation to individuals with access to and familiarity with technology. It should also be noted that the data were collected at a single point in time, making it impossible to detect changes in the assessment of these individuals’ quality of life over time. Therefore, longitudinal studies are recommended to monitor the evolution of quality of life and symptoms post-COVID-19.

Another limitation relates to the lack of investigation into correlations between participants’ clinical conditions and quality of life. Also noteworthy is the low number of confirmed respiratory comorbidities (chronic obstructive pulmonary disease, asthma, chronic bronchitis), indicating that the results were purely random.

### Contributions to health and nursing

The contributions of this study to nursing and public health lie in the fact that the distributions presented here provoke broad reflections on the effects of COVID-19 on people’s quality of life, in addition to providing useful reference values for researchers, healthcare professionals, and public managers. This information allows for the efficient and systematic conduct of studies on therapeutic interventions and prevention to mitigate the adverse effects on the physical and mental health of the population affected by COVID-19.

## CONCLUSIONS

It can be concluded that, in quality of life assessment according to the SF-36, the bodily pain domain presented the best scores and the role limitations due to personal or emotional problems domain the worst, in both the case and control groups. In the assessment using the SGRQ, the activity domain presented the worst scores and the symptoms domain the best quality of life scores. Furthermore, a direct correlation was found between the SF-36 domains, a direct correlation between the SGRQ domains, and an inverse correlation between the domains of the two questionnaires.

People with respiratory complications resulting from post-COVID-19 syndrome (case group) had a worse quality of life compared to people who had COVID-19 and did not develop respiratory complications (control group).

The findings reinforce the need for multidisciplinary follow-up strategies and the leading role of nursing in the early identification and management of respiratory sequels of post-COVID-19 syndrome, aiming to improve the quality of life of people who developed this disease.

Therefore, it is suggested that the nursing team be trained to act perceptively in identifying changes in the quality of life of these people affected by COVID-19 and in providing the necessary adaptations, within their field of action, to mitigate the effects of these complications, since nursing acts in disease prevention, health, comfort and well-being promotion for the population.

## Data Availability

The research data are available within the article.

## References

[1] National Institute for Health and Care Excellence (2020). COVID-19 Guideline: Management of the Long-Term Effects of COVID-19.

[2] Aranda J, Oriol I, Martín M, Feria L, Vázquez N, Rhyman N. (2021). Long-term impact of COVID-19 associated acute respiratory distress syndrome. J Infect.

[3] Toufen C, Pêgo-Fernandes PM. (2021). COVID-19: long-term respiratory consequences. Sao Paulo Med J.

[4] Mill JG, Polese J. (2023). Síndrome pós-COVID ou COVID Longa: um novo desafio para o sistema de saúde. Arq Bras Cardiol.

[5] Ida FS, Silva GP, Almeida IC (2024). Síndrome pós-COVID-19: sintomas persistentes, impacto funcional, qualidade de vida, retorno laboral e custos indiretos: estudo prospectivo de casos 12 meses após a infecção. Cad Saúde Pública.

[6] Pereira LB, Rodrigues JPV, Varallo FR, Pereira LRL. (2023). Qualidade de vida relacionada à saúde e resultados em longo prazo após Covid-19 levemente sintomático: explorando o protocolo. Arq Bras Cardiol.

[7] Delbressine JM, Machado FVC, Goërtz YMJ, Spruit MA, Vaes AW. (2021). The impact of post-COVID-19 syndrome on self-reported physical activity. Int J Environ Res Public Health.

[8] Ramos Junior AN (2024). Desafios da COVID longa no Brasil: uma agenda inacabada para o sistema único de saúde. Cad Saúde Pública.

[9] Davis HE, McCorkell L, Vogel JM, Topol EJ. (2023). Long COVID: major findings, mechanisms and recommendations. Nat Rev Microbiol.

[10] Muraro AP, Moreira MR, Lima LD, Andrade CLT, Carvalho MS. (2023). Óbitos por condições de saúde posteriores à COVID-19 no Brasil. Ciênc Saúde Colet.

[11] Greenhalgh T, Knight M, A’Court C, Buxton M, Husain L. (2020). Management of post-acute COVID-19 in primary care. BMJ.

[12] Scordo KA, Richmond MM, Munro N. (2021). Post-COVID-19 syndrome: theoretical basis, identification, and management. Adv Crit Care.

[13] Carvalho ACR, Martins RF, Gama JC, Marta CB, Goulart MCL, Nassar PRB. (2021). The quality of life of intensive nurses through instrument SF-36. Rev Online Pesq Cuid Fundam.

[14] Ciconelli RM. (1997). Translation to Portuguese and validation of the generic quality of life assessment questionnaire “Medical Outcomes Study 36-Item Short-Form Health Survey (SF-36)”.

[15] Abrams EM, Szefler SJ. (2020). COVID-19 and the impact of social determinants of health. Lancet Respir Med.

[16] Petersen MS, Kristiansen MF, Hanusson KD, Danielsen ME, Steig BA, Gaini S (2021). Long COVID in the Faroe Islands: a longitudinal study among non-hospitalized patients. Clin Infect Dis.

[17] Gutiérrez MAG, Ruvalcaba JCL. (2020). La salud y sus determinantes, promoción de la salud y educación sanitaria. JONNPR.

[18] Mendonça LBA. (2022). Avaliação da qualidade de vida de pessoas com complicações respiratórias na síndrome pós-Covid-19.

[19] Von Elm E, Altman DG, Egger M, Pocock SJ, Gøtzsche PC, Vandenbroucke JP, STROBE Initiative (2014). The Strengthening the Reporting of Observational Studies in Epidemiology (STROBE) statement: guidelines for reporting observational studies. Int J Surg.

[20] Fontenele MGM. (2021). Development and evaluation of software for monitoring patients diagnosed with COVID-19 and other respiratory syndromes.

[21] Hirt B, Niece SP, Moreira LB. (2022). Quality of life: analysis of functional, social and emotional aspects in a population with severe visual loss. Rev Med Paraná.

[22] Sousa TC, Jardim JR, Jones P. (2000). Validation of the Saint George’s Respiratory Questionnaire in patients with chronic obstructive pulmonary disease in Brazil. J Pneumol.

[23] Cardoso MFPT, Martins MMFPS, Trindade LL, Ribeiro OMPL, Fonseca EF. (2021). The COVID-19 pandemic and nurses’ attitudes to ward death. Rev Latino-Am Enfermagem.

[24] Matioli MR. (2021). The health-related quality of life and emotional aspects of COVID-19 survivors after discharge from the Intensive Care Unit, from the perspectives of Lacanian psychoanalysis and health sciences.

[25] Rocha RPS, Silva NDC, Santos MSS, Teixeira JJB, Lopes JM, Cavalcante LPM (2024). Síndrome pós-COVID-19 entre hospitalizados por COVID-19: estudo de coorte após 6 e 12 meses da alta hospitalar. Cad Saúde Pública.

[26] Castro AR, Dores CC, Freitas FAS, Castro FM, Porto APM, Moreira FJF. (2022). Symptomatological investigation and complications by Covid-19 of post-admission patients between 2020 and 2021 in a Brazilian state. Saude (Santa Maria).

[27] Jimeno-Almazán A, Pallarés JG, Buendía-Romero A, Martínez-Cava A, Franco-López F, Martínez BJS (2021). Post-COVID-19 syndrome and the potential benefits of exercise. Int J Environ Res Public Health.

[28] Davis HE, Assaf GS, McCorkell L, Wei H, Low RJ, Re’em Y (2021). Characterizing long COVID in an international cohort: 7 months of symptoms and their impact. EClinicalMedicine.

[29] Rahmati M, Udeh R, Yon DK, Lee SW, Dolja-Gore X, McEvoy M (2023). A systematic review and meta-analysis of long-term sequelae of COVID-19 2-year after SARS-CoV-2 infection: a call to action for neurological, physical, and psychological sciences. J Med Virol.

[30] Fantacini CMF. (2019). Social determinants of mental health in older adults.

[31] Anastasio F, Barbuto S, Scarnecchia E, Cosma P, Fugagnoli A, Rossi G (2021). Medium-term impact of COVID-19 on pulmonary function, functional capacity and quality of life. Eur Respir J.

[32] Silva FMS, Gomes JAC, Chaves PHN. (2022). Independence in AVDs and quality of life in elderly survivors of COVID-19 in the rural area of Coroatá-MA. Rev Pesq Fisioter.

[33] Silva RO, Pereira JN, Milan EGP. (2021). Quality of life assessment with the SF-36 instrument during the COVID-19 pandemic: a pilot study. Res Soc Dev.

[34] Almeida SM, Andrade CAS, Castro JSM, Almeida CS, Almeida AC. (2021). Work-related covid-19 cases at the state of Bahia: epidemiological profile. Rev Baiana Saude Publica.

[35] Pereira C, Harris BHL, Di Giovannantonio M, Rosadas C, Short C, Quinlan R (2021). The association between antibody response to Severe Acute Respiratory Syndrome Coronavirus 2 infection and post-COVID-19 syndrome in health care workers. J Infect Dis.

[36] Sirayder U, Inal-Ince D, Kepenek-Varol B, Acik C. (2022). Long-term characteristics of severe COVID-19: respiratory function, functional capacity, and quality of life. Int J Environ Res Public Health.

[37] Biswas A, Pandey S, Ghosh S, Pandey J, Kumar N, Das S (2022). Factors associated with persistence of dyspnea and change in health-related quality of life in patients with COVID-19 after discharge. Cureus.

[38] Del Rio C, Collins LF, Malani P. (2020). Long-term health consequences of COVID-19. JAMA.

