# Supplementary material for: Quality of life of people with and without respiratory complications in post-COVID-19 syndrome: a retrospective study
Source: Rev Bras Enferm. 2026 Aug 21;79:e20250195. doi: 10.1590/0034-7167-2025-0195 (PMC13506210; doi:10.1590/0034-7167-2025-0195)
Supplement: Translation

# Translation: Qualidade de vida de pessoas com e sem complicações respiratórias na síndrome pós-COVID-19: estudo retrospectivo

**DOI:** 10.1590/0034-7167-2025-0195pt

**Authors:** Larissa Bento de Araújo Mendonça, Francisca Elisangela Teixeira Lima, Lívia Maia Pascoal, Paulo César de Almeida, Patricia Neyva da Costa Pinheiro, Glaubervania Alves Lima, Maria Gabriela Miranda Fontenele, Lorena Pinheiro Barbosa

**Affiliations:** I Universidade Federal do Ceará. Fortaleza, Ceará, Brasil; II Universidade Federal do Maranhão. Imperatriz, Maranhão, Brasil; III Universidade Estadual do Ceará. Fortaleza, Ceará, Brasil

**Keywords:** Qualidade de Vida, Síndrome de Pós-COVID-19 Aguda, Estudos Retrospectivos, Infecções Respiratórias, Enfermagem.

## Abstract

**Objetivos:**

avaliar e comparar a qualidade de vida (QV) de pessoas com e sem complicações respiratórias decorrentes da síndrome pós-COVID-19.

**Métodos:**

estudo observacional retrospectivo, comparativo e analítico, com abordagem quantitativa e amostragem não probabilística por conveniência, desenvolvido com 300 pessoas residentes no Ceará, Maranhão ou Pernambuco, distribuídas em grupo caso (n=100/com complicações respiratórias) e controle (n=200/sem complicações respiratórias). Para coleta (2021-2022), utilizaram-se o *Medical Outcomes Study 36-Item Short-Form Health Survey* (SF-36) e o *St George’s Respiratory Questionnaire* (SGRQ). Realizaram-se os testes t de Student, ANOVA e correlação de Pearson (p<0,05).

**Resultados:**

grupo caso apresentou escores menores em todos os domínios do SF-36 (p<0,05), indicando pior QV. No SGRQ, sintomas, atividade e impactos psicossociais apresentaram maior comprometimento (p<0,001). Observou-se correlação linear inversa entre os escores do SF-36 e SGRQ.

**Conclusões:**

pessoas com complicações respiratórias pós-COVID-19 apresentaram pior QV, evidenciando a relevância do acompanhamento multiprofissional na reabilitação respiratória para melhoria da QV.

## INTRODUÇÃO

Disfunções respiratórias figuram entre as complicações mais prevalentes entre pessoas que desenvolveram a síndrome pós-COVID-19, condição caracterizada pela persistência de sinais e sintomas da doença por mais de quatro semanas após a infecção aguda. Esses sintomas incluem tosse, fadiga, dispneia, cefaleia, dificuldades neurocognitivas, alterações mentais, anosmia, disgeusia, entre outros, afetando negativamente a qualidade de vida das pessoas acometidas^(1-6)^.

Entre os sintomas persistentes mais associados à síndrome, destaca-se a dispneia. Nos Estados Unidos, estudo identificou esse sintoma como um dos mais frequentemente relatados por participantes com síndrome pós-COVID-19, associando-se a prejuízos na qualidade de vida e ao aumento da dependência de terceiros para a realização de atividades de autocuidado^(7)^. No Brasil, considerando uma prevalência de 10%, estima-se que aproximadamente 4 milhões de brasileiros desenvolveram essa síndrome^(8-10)^.

Muitos pacientes que desenvolvem a síndrome pós-COVID-19 relataram sintomas persistentes que limitaram a sua capacidade de trabalho e participação social. Pessoas anteriormente saudáveis passaram a apresentar sintomas inesperados, enquanto aquelas com comorbidades prévias enfrentaram o agravamento de suas condições clínicas, o que contribuiu para o comprometimento da funcionalidade e qualidade de vida^(11,12)^.

A qualidade de vida, enquanto conceito multidimensional, refere-se à percepção do indivíduo sobre sua posição na vida, considerando o contexto cultural e o sistema de valores em que está inserido, bem como seus objetivos, expectativas, padrões e preocupações. Ademais, está diretamente relacionada aos diversos fatores da vida humana, tais como saúde, condição financeira, trabalho, família, meio ambiente, lazer, entre outros^(13)^.

Nesse contexto, a avaliação da qualidade de vida de pessoas com síndrome pós-COVID-19 é essencial para compreender o impacto das alterações respiratórias decorrentes da infecção. Para tanto, diferentes instrumentos têm sido propostos na literatura para avaliar esse desfecho, tanto em populações gerais quanto em grupos com doenças específicas^(14)^.

Diante dos impactos globais da COVID-19 na saúde e na funcionalidade das populações, torna-se fundamental estudar e compreender a síndrome pós-COVID-19 em sua dimensão clínica e social, bem como prever seu impacto cumulativo, monitorar taxas de hospitalização e mortalidade, e estimar sua prevalência^(15,16)^. Embora a literatura global aborde de forma mais ampla as sequelas pós-COVID-19, ainda existe uma lacuna de estudos que investiguem a qualidade de vida e as complicações respiratórias em diferentes contextos regionais do Brasil, especialmente no Nordeste, no qual as particularidades socioeconômicas e de acesso aos serviços de saúde podem influenciar esses desfechos.

Assim, o levantamento de dados sobre a qualidade de vida de pessoas que desenvolveram complicações respiratórias decorrentes da síndrome pós-COVID-19 fornecerá informações que poderão subsidiar o planejamento de programas de prevenção de doenças e promoção da saúde, considerando as influências micro e macrossociais no contexto de ações em saúde^(17)^. Hipotetiza-se que pessoas com complicações respiratórias oriundas da síndrome pós-COVID-19 apresentem pior qualidade de vida em comparação com aquelas sem complicações.

### Relevância do estudo

A relevância deste estudo reside em evidenciar o impacto das condições respiratórias pós-COVID-19 na qualidade de vida de pessoas residentes no Nordeste do Brasil, região marcada por desigualdades sociais e desafios históricos de acesso aos serviços de saúde. A síndrome pós-COVID-19 tem gerado crescente demanda por cuidados de saúde, especialmente entre pessoas que desenvolvem complicações respiratórias persistentes. Entretanto, ainda são escassas as investigações regionais que avaliem, de forma comparativa, o impacto dessas complicações na qualidade de vida, sobretudo no Nordeste brasileiro, onde desigualdades socioeconômicas e de acesso aos serviços podem intensificar esse comprometimento. Os resultados deste estudo oferecem evidências que podem orientar ações de vigilância em saúde, práticas de reabilitação respiratória e estratégias multiprofissionais de acompanhamento, contribuindo para o planejamento de políticas públicas e para a qualificação da assistência à população afetada.

Esse artigo é produto de tese^(18)^ depositada em repositório institucional: http://repositorio.ufc.br/handle/riufc/70986.

## OBJETIVOS

Avaliar e comparar a qualidade de vida de pessoas com e sem complicações respiratórias decorrentes da síndrome pós-COVID-19.

## MÉTODOS

### Aspectos éticos

O estudo foi aprovado pelo Comitê de Ética em Pesquisa, em conformidade com os preceitos éticos da Resolução n^o^ 466/2012 do Conselho Nacional de Saúde, sob Parecer nº 4.278.495/2020 e Certificado de Apresentação de Apreciação Ética n^o^ 36193820.1.0000.5054.

### Tipo de estudo

Trata-se de estudo observacional retrospectivo, de natureza comparativa e analítica, com abordagem quantitativa, desenvolvido em três estados do Nordeste brasileiro: Ceará, Maranhão e Pernambuco. As recomendações para os estudos observacionais foram seguidas conforme o *STrengthening the Reporting of OBservational studies in Epidemiology* ^(19)^.

### População, amostra e critérios de seleção

A população do estudo foi composta por pessoas que foram diagnosticadas com COVID-19, cujos dados foram inicialmente identificados no Sistema de Informação da Vigilância Epidemiológica da Gripe (SIVEP-GRIPE) e no sistema e-SUS Notifica. A partir desta população, a amostra selecionada foi organizada em dois grupos: grupo caso e grupo controle. O grupo caso foi formado por pessoas com idade > 18 anos, que apresentaram complicações respiratórias durante a doença com permanência de, pelo menos, quatro semanas após o diagnóstico da COVID-19, caracterizando a síndrome pós-COVID-19, e que adoeceram entre março de 2020 e janeiro de 2022. O grupo controle foi composto por pessoas que atenderam aos mesmos critérios do grupo caso, exceto ter apresentado complicações respiratórias decorrentes da síndrome pós-COVID-19.

Foram excluídos os participantes que não responderam completamente aos questionários, que os responderam após o término da coleta de dados ou que não apresentavam condições cognitivas para responder aos questionários, totalizando 27 exclusões. O manejo de dados faltantes foi realizado por exclusão *listwise*, mantendo-se apenas os questionários totalmente válidos na análise final.

O cálculo amostral foi baseado na fórmula para estudos de caso-controle (n= z^2^_5%_ {1/[ P_1_(1 - P_1_)] + 1/[P_2_(1 - P_2_)]} / [log_e_(1- ɛ)]^2^), considerando-se uma razão de chances de 2, probabilidade encontrada de 10% de pacientes com doença pulmonar obstrutiva crônica, precisão relativa de 50% e nível de significância de 10%, resultando em 100.

Com o intuito de fornecer uma estimativa mais precisa da frequência de exposição no grupo controle e aumentar o poder do estudo, aumentando a capacidade de detectar uma associação, se ela está realmente estiver presente, optou-se por utilizar o dobro da amostra calculada para o grupo controle, totalizando 200 pessoas.

### Protocolo do estudo

Primeiramente, os dados foram coletados no SIVEP-GRIPE e no e-SUS Notifica, os quais contêm a ficha de notificação de casos de pacientes com COVID-19 e compiladas pela Vigilância Epidemiológica da Secretaria Estadual de Saúde (SESA) dos estados do Ceará, Maranhão e Pernambuco. A autorização para acesso e uso dos dados foi obtida mediante solicitação formal às respectivas SESAs. Posteriormente, foi enviado, via aplicativo de mensagens instantâneas, um *link* para o participante acessar um formulário e preenchê-lo, caso aceitasse participar da pesquisa. Em ambos os grupos, foi aplicado um formulário validado de caracterização das pessoas que tiveram COVID-19^(20)^, contendo perguntas sobre as características sociodemográficas e clínicas dos participantes da pesquisa e o *Medical Outcomes Study 36-Item Short-Form Health Survey* (SF-36), validado para uso no Brasil^(14)^. O *Saint George’s Respiratory Questionnaire* (SGRQ) foi aplicado apenas aos que afirmaram ter tido complicações respiratórias, com o objetivo de avaliar o impacto na qualidade de vida desse grupo.

O questionário de qualidade de vida SF-36 avalia a qualidade de vida dos participantes referente às quatro últimas semanas. É composto por 36 itens, divididos em oito domínios: 1 -capacidade funcional (questão nº 3, com dez itens); 2 - limitação por aspectos físicos (questão nº 4, com quatro itens); 3 - dor (questões nº 7, com um item, e nº 8, com um item); 4 - estado geral de saúde (questões nº 1, com um item, e nº 11, com quatro itens); 5 - vitalidade (questão nº 9, com quatro itens correspondentes às letras: a + e + g + i); 6 - aspectos sociais (questões nº 6, com um item, e nº 10, com um item); 7 - limitação por aspectos emocionais (questão nº 5, com três itens); 8 - saúde mental (questão nº 9, com cinco itens representados pelas letras: b + c + d + f + h); e uma questão (questão n^o^ 2) que avalia a alteração do estado de saúde, mas que não é empregada no cálculo dos domínios, sendo aplicada somente para avaliar o quanto o indivíduo está melhor ou pior da sua saúde comparado a um ano atrás^(21)^.

Para avaliação de seus resultados, após sua aplicação, é dado um escore para cada questão, que posteriormente são transformados em uma escala de zero a 100, sendo zero o pior estado de saúde, e 100, o melhor. Cada dimensão é analisada separadamente^(14)^.

O SGRQ, utilizado no grupo caso, é composto por três domínios: sintomas; atividade; e impactos psicossociais. O valor do domínio sintomas é calculado somando-se todas as pontuações das questões da parte 1 do questionário, cuja pontuação de cada questão pode variar de zero a 93,3, dividindo-se o valor por 662,5. O domínio atividade é calculado somando-se todas as pontuações das seções 2 e 6 da parte 2 do questionário, cuja pontuação de cada questão pode variar de zero a 90,6, dividindo-se o valor por 1.209,1. E o domínio impactos psicossociais é calculado somando-se todas as pontuações das seções 1, 3, 4, 5 e 7 do questionário 2, cuja pontuação de cada questão pode variar de zero a 96,7, dividindo-se o valor por 2.117,8. O total é referido como um percentual máximo. Valores próximos de zero representam uma melhor qualidade de vida naquele domínio^(22)^.

Ressalta-se que a aplicação do SGRQ foi realizada apenas no grupo caso, por se tratar de um instrumento específico para avaliar a qualidade de vida de pessoas com sintomas respiratórios crônicos. As análises envolvendo o SGRQ se limitaram à descrição dos escores dentro do grupo caso e à análise de correlação com os domínios do SF-36, que também foi aplicado a esse grupo.

A coleta de dados ocorreu durante os meses de novembro de 2021 a abril de 2022. Inicialmente, os dados foram coletados nas fichas de notificação de casos de COVID-19 inseridas e disponíveis nos sistemas de notificação do Ministério da Saúde do Brasil (SIVEP-GRIPE e e-SUS Notifica) dos estados do Ceará, Maranhão e Pernambuco. Conforme liberação prévia pela SESA de cada estado, os dados foram exportados para o *software* de monitoramento de COVID-19 e síndromes respiratórias, desenvolvido e validado no Brasil^(20)^.

Após, o *software* gerou um *link,* o qual foi enviado, pelos próprios pesquisadores, para as pessoas com COVID-19 previamente cadastradas, por meio de aplicativo de mensagens. O *link* era acompanhado da seguinte pergunta: você teve COVID-19 e reside nos estados do Ceará, Maranhão ou Pernambuco? Os participantes que responderam positivamente à pergunta, ao clicar no *link*, obtiveram acesso ao Termo de Consentimento Livre e Esclarecido. Após aceitar participar da pesquisa, eram automaticamente direcionados para o preenchimento do formulário de caracterização e do SF-36. Os participantes que relataram ter desenvolvido complicações respiratórias decorrentes da COVID-19 foram alocados no grupo caso e, além dos instrumentos mencionados anteriormente, também foram direcionados para o preenchimento do SGRQ.

A confiabilidade dos dados foi controlada por meio de vinculação do formulário eletrônico ao CPF, número de telefone e endereço de e-mail previamente cadastrados nos sistemas oficiais de notificação (SIVEP-GRIPE e e-SUS Notifica), o que impediu respostas duplicadas e permitiu verificar a identidade dos participantes.

### Análise dos resultados e estatística

Os dados foram processados e analisados no *Statistical Package for the Social Sciences*, versão 20.0, sob licença 10101131007. A normalidade das variáveis foi verificada pelo teste de Kolmogorov-Smirnov. As médias foram comparadas pelos testes paramétricos t de Student e ANOVA. As associações entre variáveis categóricas foram analisadas por meio do teste qui-quadrado ou teste exato de Fisher. Foram consideradas como estatisticamente significativas as análises inferenciais com p<0,05.

## RESULTADOS

As características sociodemográficas, clínicas e estilo de vida foram avaliadas quanto à homogeneidade dos grupos e não apresentaram diferenças significativas (p>0,05), ou seja, ambos os grupos eram semelhantes entre si, conforme consta na Tabela 1.

**Tabela 1:** Distribuição das pessoas com COVID-19 segundo as características sociodemográficas, clínicas e estilo de vida no grupo caso e grupo controle (N=300), Fortaleza, Ceará, Brasil, 2022

Variáveis	Grupo caso		Grupo controle		Valor de *p* ^*^
	N=100	%	N=200	%	
Idade					0,410
<60	95	95	187	93,5	
>60	5	5	13	6,5	
**Sexo**					0,557
Feminino	64	64	121	60,5	
Masculino	36	36	79	39,5	
**Escolaridade**					0,828
Até ensino médio	19	19	45	22,5	
Ensino superior	71	71	134	67	
Pós-graduação	10	10	21	10,5	
**Profissão**					0,508
Profissional de saúde	24	24	47	23,5	
Profissional de outras áreas	50	50	112	56	
Outros	26	26	41	20,5	
**Estado civil**					0,190
Solteiro	42	42	87	43,5	
Casado/união estável	52	52	88	44	
Divorciado/separado/viúvo	6	6	25	12,5	
**Estado em que reside**					0,810
Ceará	71	71	128	64	
Maranhão	27	27	47	23,5	
Pernambuco	2	2	25	12,5	
**Condições clínicas antes da COVID-19**					
Alergias respiratórias	23	23	36	18	0,304
Diabetes	5	5	15	7,5	0,413
Doença pulmonar	4	4	0	0	0,098
Hipertensão arterial	16	16	23	11,5	0,275
Obesidade	10	10	12	6	0,210
Sobrepeso	16	16	32	16	1,000
Nenhuma	41	41	105	52,5	
**Sintomas da COVID-19**					
Adinamia/fadiga	24	24	20	10	0,352
Anosmia	26	26	76	38	0,052
Cefaleia	46	46	91	45,5	0,935
Coriza/catarro	22	22	44	22	1,000
Disgeusia	27	27	67	33,5	0,253
Dispneia	18	18	22	11	0,490
Dor de garganta	30	30	51	25,5	0,408
Dor no corpo	49	49	55	27,5	0,134
Febre	46	46	96	48	0,744
Tosse	29	29	56	28	0,856
Outros	37	37	53	26,5	0,059
Sem sintomas	1	1	15	7,5	
**Desenvolveu complicações na síndrome pós-COVID-19 (exceto respiratórias)**					0,377
Sim	75	75	87	43,5	
Não	25	25	113	56,5	
**Tipos de complicações desenvolvidas**					
Adinamia/fadiga	31	31	15	7,5	0,587
Anosmia	7	7	10	5	0,480
Ansiedade	11	11	7	3,5	0,085
Cefaleia	8	8	9	4,5	0,216
Déficit de concentração	5	5	8	4	0,388
Déficit de memória	10	10	19	9,5	0,890
Insônia	6	6	3	1,5	0,069
Queda de cabelo	22	22	21	10,5	0,274
**Prática de atividade física**					0,412
Sim	51	51	161	80,5	
Não	49	49	39	19,5	
**Tabagismo**					1,00
Sim	4	4	9	4,5	
Não	96	96	191	95,5	
**Etilismo**					0,406
Sim	44	44	78	39	
Não	56	56	122	61	

* *Teste qui-quadrado ou teste exato de Fisher.*

A maioria dos participantes, de ambos os grupos (caso e controle), tinha idade inferior a 60 anos (média de 35,95 + 12,1 anos), era do sexo feminino, possuía ensino superior (completo ou incompleto), não era trabalhador da área da saúde, era casado/união estável e residia no estado do Ceará.

As condições clínicas preexistentes mais comuns incluíram alergias respiratórias, sobrepeso e hipertensão arterial sistêmica (HAS), enquanto dor no corpo, febre e cefaleia foram os sintomas da COVID-19 mais relatados. Adinamia/fadiga, queda de cabelo e ansiedade foram as manifestações da síndrome pós-COVID-19 que mais se destacaram, com tosse persistente, dispneia e cansaço aos esforços físicos sendo as complicações respiratórias prevalentes no Grupo. O estilo de vida antes da COVID-19 foi caracterizado pela prática de atividade física regular e baixos índices de tabagismo e etilismo.

Na avaliação da qualidade de vida pelo SF-36, os participantes com complicações respiratórias (grupo caso) apresentaram médias significativamente menores em todos os oito domínios em comparação aos pacientes sem essas complicações (grupo controle), indicando uma pior qualidade de vida no grupo caso. As diferenças encontradas foram clinicamente e estatisticamente significativas em todos os domínios, conforme demonstrado na Tabela 2. Destaca-se que os maiores impactos negativos foram identificados nos domínios de dor, aspectos físicos e capacidade funcional.

**Tabela 2:** Comparação das médias dos domínios do *Medical Outcomes Study 36-Item Short-Form Health Survey*, desvio padrão e valor de p nos grupos caso e grupo controle (N=300), Fortaleza, Ceará, Brasil, 2022

Domínios	Grupo	n	Média (+DP)	Diferença de média	Valor de *p* ^*^
**Capacidade funcional**	Caso	100	45,3(+18,2)	-7,9	**<0,001**
	Controle	200	53,2(+15,9)		
**Aspectos físicos**	Caso	100	29,3(+21,1)	-9,8	**<0,001**
	Controle	200	39,1(+17,2)		
**Dor**	Caso	100	51,7(+19,7)	-11,3	**<0,001**
	Controle	200	63,0(+18,6)		
**Estado geral da saúde**	Caso	100	47,8(+15,9)	-6,2	**<0,001**
	Controle	200	54,0(+14,3)		
**Vitalidade**	Caso	100	39,6(+18,1)	-6,9	**<0,001**
	Controle	200	46,5(+17,3)		
**Aspectos sociais**	Caso	100	48,8(+22,0)	-6,6	**0,010**
	Controle	200	55,4(+20,2)		
**Aspectos emocionais**	Caso	100	29,0(+22,0)	-6,0	**0,021**
	Controle	200	35,0(+20,0)		
**Saúde mental**	Caso	100	45,4(+17,0)	-7,8	**<0,001**
	Controle	200	53,2(+15,9)		

* *Teste t de Student; DP - desvio padrão.*

No grupo caso, a avaliação dos domínios do SGRQ revelou que o domínio sintomas apresentou pontuações que variam de zero a 88,1; o domínio atividade variou de zero a 100; e o domínio impactos psicossociais variou de zero a 99,3. A média do domínio sintomas (33,2) foi a menor em comparação com os domínios impactos psicossociais (41,2) e atividade (57,6), com diferença estatística em todos os domínios (p<0,001) (Tabela 3), evidenciando uma melhor qualidade de vida no domínio sintomas e uma pior no domínio atividade.

**Tabela 3:** Análise das médias, desvio padrão e valor de p dos domínios do *St George’s Respiratory Questionnaire* no grupo caso (N=100), Fortaleza, Ceará, Brasil, 2022

Domínios do SGRQ (N=100)	Média (+DP)	Valor de *p* ^*^
Sintomas	33,2(+23,1)	**<0,001**
Atividade	57,6(+34,3)	**<0,001**
Impactos psicossociais	41,2(+27,4)	**<0,001**

DP - desvio padrão; SGRQ - St George’s Respiratory Questionnaire;

* ANOVA p<0,001.

Na análise de correlação linear (Tabela 4), observou-se que todos os domínios do SGRQ se correlacionaram entre si de maneira diretamente proporcional (p<0,001). De forma semelhante, os oito domínios do SF-36 se correlacionaram entre si de maneira diretamente proporcional (p<0,001).

**Tabela 4:** Análise de correlação linear entre os domínios do *Medical Outcomes Study 36-Item Short-Form Health Survey* e do *St George’s Respiratory Questionnaire* nos grupos caso e grupo controle (N=300), Fortaleza, Ceará, Brasil, 2022

		Domínios do SGRQ		Domínios do SF-36							
		Atividade	Impactos psicossociais	Capacidade funcional	Aspectos físicos	Dor	Estado geral da saúde	Vitalidade	Aspectos sociais	Aspectos emocionais	Saúde mental
**Domínios do SGRQ**											
Sintomas	r	0,679	0,732	-0,207	-0,219	-0,148	-0,211	-0,109	-0,195	-0,095	-0,178
	p	**<0,001**	**<0,001**	**0,039**	**0,028**	0,141	**0,035**	0,280	0,052	0,346	0,077
Atividade	r		0,836	-0,368	-0,514	-0,428	-0,330	-0,398	-0,346	-0,277	-0,341
	p		**<0,001**	**<0,001**	**<0,001**	**<0,001**	**0,001**	**<0,001**	**<0,001**	**0,005**	**0,001**
Impactos psicossociais	r			-0,355	-0,514	-0,363	-0,325	-0,361	-0,424	-0,361	-0,350
	p			**<0,001**	**<0,001**	**<0,001**	**0,001**	**<0,001**	**<0,001**	**<0,001**	**<0,001**
**Domínios do SF-36**											
Capacidade funcional	r				0,500	0,513	0,323	0,488	0,521	0,296	0,406
	p				**<0,001**	**<0,001**	**<0,001**	**<0,001**	**<0,001**	**<0,001**	**<0,001**
Aspectos físicos	r					0,539	0,288	0,476	0,501	0,529	0,372
	p					**<0,001**	**<0,001**	**<0,001**	**<0,001**	**<0,001**	**<0,001**
Dor	r						0,414	0,483	0,520	0,341	0,376
	p						**<0,001**	**<0,001**	**<0,001**	**<0,001**	**<0,001**
Estado geral da saúde	r							0,531	0,436	0,330	0,581
	p							**<0,001**	**<0,001**	**<0,001**	**<0,001**
Vitalidade	r								0,673	0,505	0,759
	p								**<0,001**	**<0,001**	**<0,001**
Aspectos sociais	r									0,561	0,646
	p									**<0,001**	**<0,001**
Aspectos emocionais	r										0,497
	p										**<0,001**

*r - Coeficiente de Correlação de Pearson; p - valor de significância do teste; SGRQ - St George’s Respiratory Questionnaire; SF-36 - Medical Outcomes Study 36-Item Short-Form Health Survey.*

Em relação à correlação entre os domínios do SGRQ e do SF-36, o domínio sintomas do SGRQ se correlacionou de maneira inversamente proporcional com três domínios do SF-36: capacidade funcional (p=0,039); aspectos físicos (p=0,028); e estado geral de saúde (p=0,035). Enquanto isso, os domínios atividade e impactos psicossociais do SGRQ se correlacionaram de maneira inversamente proporcional com todos os oito domínios do SF-36 (p<0,0001).

Esses achados demonstram que as escalas apresentaram uma correlação inversamente proporcional estatisticamente significativa, denotando que, quanto maiores as médias alcançadas nos domínios do SF-36, menor a pontuação alcançada nos domínios do SGRQ, demonstrando que os domínios dos questionários se correlacionam entre si e apresentam interpretações semelhantes em termos de análise de escore total. Para o SF-36, quanto maior o escore obtido naquele domínio, melhor é a qualidade de vida do indivíduo, enquanto que, no SGRQ, a qualidade de vida naquele domínio é melhor à medida que os escores diminuem.

## DISCUSSÃO

Este estudo investigou o impacto da síndrome pós-COVID-19 na qualidade de vida de indivíduos residentes no Nordeste do Brasil, com foco nas complicações respiratórias.

O caráter regional deste estudo, realizado em três estados do Nordeste brasileiro, amplia a representatividade dos achados e contribui para a literatura nacional e latino-americana, ao evidenciar particularidades socioculturais e desafios de acesso aos serviços de saúde que influenciam a qualidade de vida de pessoas acometidas pela síndrome pós-COVID-19.

As características sociodemográficas dos participantes, majoritariamente adultos jovens, do sexo feminino, com ensino superior e ativos profissionalmente, convergem com perfis identificados em estudos realizados em Portugal e no Brasil (Ribeirão Preto, São Paulo)^(23,24)^. Essa consistência sugere que, embora a infecção possa afetar todas as faixas etárias, populações mais jovens e ativas também enfrentam sequelas significativas, desafiando a percepção inicial de que apenas idosos seriam gravemente impactados.

As comorbidades referidas neste estudo corroboram os achados de pesquisa conduzida na Dinamarca que investigou pacientes diagnosticados com síndrome pós-COVID-19, constatando que 87,5% deles apresentavam alguma comorbidade pré-existente, predominando HAS, enfisema, asma, colite ulcerativa, diabetes e doenças cardiovasculares^(16)^. Estudos brasileiros também identificaram a HAS e o pré-diabetes/diabetes como comorbidades prévias frequentes^(5,25)^.

Pesquisa realizada no Brasil com 470 pessoas acometidas pela COVID-19 constatou alta prevalência de sintomas respiratórios, ocupando a terceira posição entre os sintomas mais relatados pelos pacientes, superados apenas por fadiga e alopecia^(26)^. Entre as complicações respiratórias da síndrome pós-COVID-19, a dispneia é um dos sintomas mais prevalentes, conforme evidenciado por estudos realizados em diferentes países^(8,27-29)^.

Ressalta-se que a prevalência de comorbidades pré-existentes e a gravidade da infecção inicial podem atuar como fatores confundidores na avaliação da qualidade de vida pós-COVID-19. A ausência de uma estratificação mais detalhada quanto à gravidade da infecção e a confirmação da presença de comorbidades respiratórias confirmadas na análise de caso-controle podem ter atenuado a força das associações encontradas.

O estilo de vida saudável adotado pelos participantes desta pesquisa também foi observado em outro estudo, reforçando que a prática regular de atividade física, alimentação balanceada, abstinência do fumo e álcool, e uso adequado de medicamentos contribuem para a prevenção de doenças, declínio funcional, longevidade e melhor qualidade de vida^(30)^.

Neste estudo, a qualidade de vida das pessoas com complicações respiratórias decorrentes da síndrome pós-COVID-19 (grupo caso) foi inferior à daquelas sem tais complicações (grupo controle). Esse resultado pode ser justificado pelo comprometimento do sistema respiratório durante a COVID-19, decorrente principalmente da resposta imune exacerbada do hospedeiro e lesão inflamatória de órgãos na fase aguda da doença. Testes de função pulmonar demonstraram redução da capacidade vital forçada, prejuízo nas trocas gasosas e redução da força muscular respiratória, com necessidade de reabilitação respiratória^(31)^.

Estudo com 20 idosos diagnosticados com COVID-19 evidenciou escores inferiores a 60 pontos em todos os domínios do SF-36, sugerindo baixa qualidade de vida^(32)^. Por outro lado, pesquisa realizada em Minas Gerais com 114 participantes evidenciou que a maioria dos participantes apresentou escores superiores a 50 pontos em todos os domínios do SF-36. Os homens apresentaram melhores resultados que as mulheres nos domínios de capacidade funcional, dor, aspectos sociais e aspectos emocionais^(33)^.

No contexto dos profissionais de saúde, estudo realizado com 33 enfermeiros atuantes em Unidade de Terapia Intensiva de hospital do Rio de Janeiro revelou comprometimento físico e psicológico relevante, refletido nos domínios estado geral de saúde e vitalidade^(13)^. De maneira geral, o impacto da pandemia foi expressivo: na Bahia, estudo com 2.920 trabalhadores com COVID-19 apontou que 8,9% relataram alguma incapacidade decorrente da doença^(34)^. Outro estudo evidenciou que o maior impacto da COVID-19 foi no trabalho, visto que quase metade dos participantes estava trabalhando em horário reduzido, e 23,3% não conseguiram retornar ao trabalho^(28)^. Ainda, 16% dos indivíduos com síndrome pós-COVID-19 relataram comprometimento na prática de esportes ou atividades recreativas^(35)^.

É importante destacar que os sintomas da síndrome pós-COVID-19 podem persistir por meses, mesmo em casos leves da doença^(16)^. No presente estudo, segundo o SGRQ, o grupo caso apresentou melhor qualidade de vida no domínio sintomas e pior no domínio atividade. Esse achado condiz com estudo que também avaliou a qualidade de vida por meio do SGRQ, constatando que o grupo de 26 pessoas com complicações respiratórias da COVID-19 apresentou pior qualidade de vida em comparação com o grupo controle, formado por 26 pessoas saudáveis^(36)^.

Diferentes comorbidades são associadas à gravidade dos sintomas e ao impacto negativo na qualidade de vida. Fatores como idade, tabagismo, diabetes, hipotireoidismo e asma foram relacionados à recuperação mais lenta e melhoria da qualidade de vida^(37)^. O estigma relacionado à COVID-19 se tornou generalizado, podendo resultar em uma sensação de desesperança. Relatos crescentes de mal-estar prolongado e exaustão têm contribuído para o surgimento de transtornos emocionais, ansiedade e sofrimento psíquico, afetando consideravelmente a qualidade de vida^(38)^.

Diante desse contexto, os instrumentos de avaliação da qualidade de vida se tornam ferramentas valiosas para quantificar, em termos subjetivos e objetivos, os distúrbios de saúde percebidos pelo paciente e pelo profissional. Esses instrumentos são aplicáveis em diferentes tipos de doença, tratamentos ou intervenções, entre culturas e lugares diferentes, e podem ter suas pontuações analisadas de forma distinta^(22)^. No presente estudo, observou-se uma correlação linear entre os domínios do SF-36 e do SGRQ, no qual ambos demonstraram correlação inversamente proporcional entre si, uma vez que maiores escores no SF-36 indicam melhor qualidade de vida, enquanto que, no SGRQ, menores escores estão relacionados a melhores condições^(14,22)^.

Portanto, é imperativo que o atendimento de pessoas que desenvolveram complicações decorrente da síndrome pós-COVID-19 seja realizado com uma abordagem interdisciplinar e sustentado por uma agenda de pesquisa integrada, para evitar a fragmentação do cuidado em saúde e permitir o acompanhamento das consequências a longo prazo da síndrome pós-COVID-19^(38)^. Os achados deste estudo trazem implicações significativas para a prática de enfermagem, especialmente no âmbito da reabilitação e do cuidado continuado.

### Limitações do estudo

Como limitações, destaca-se a possibilidade de vieses de autorrelato, incluindo vieses recordatórios por parte dos participantes da pesquisa, visto que os dados foram coletados a partir de informações do passado. O uso de formulários *online* pode ter introduzido um viés de seleção, limitando a participação a indivíduos com acesso e familiaridade com a tecnologia. Ressalta-se também que os dados foram coletados em um único momento, não sendo possível detectar mudanças na avaliação da qualidade de vida dessas pessoas ao longo do tempo. Assim, recomenda-se a realização de estudos longitudinais para monitorar a evolução da qualidade de vida e dos sintomas pós-COVID-19.

Outra limitação se refere à não investigação de correlações entre condições clínicas do participante com a qualidade de vida. Destaca-se também o baixo número de casos de comorbidades respiratórias confirmadas (doença pulmonar obstrutiva crônica, asma, bronquite crônica), evidenciando que os resultados foram puramente ao acaso.

### Contribuições para a área da saúde e da enfermagem

As contribuições deste estudo para a enfermagem e para a saúde pública estão no fato de que as distribuições aqui apresentadas provocam amplas reflexões acerca dos efeitos da COVID-19 sobre a qualidade de vida das pessoas, além de fornecer valores de referência úteis para pesquisadores, profissionais de saúde e gestores públicos. Essas informações permitem conduzir de forma eficiente e sistemática estudos de intervenções terapêuticas e prevenção para atenuar os efeitos adversos à saúde física e mental da população acometida pela COVID-19.

## CONCLUSÕES

Pode-se concluir que, na avaliação da qualidade de vida segundo o SF-36, o domínio dor apresentou os melhores escores e o domínio aspectos emocionais, os piores, nos grupos caso e controle. Na avaliação pelo SGRQ, o domínio atividade apresentou piores escores e o domínio sintomas, melhores escores de qualidade de vida. Ademais, constataram-se correlação direta entre os domínios do SF-36, correlação direta entre os domínios do SGRQ e correlação inversa entre os domínios dos dois questionários.

Pessoas com complicações respiratórias decorrentes da síndrome pós-COVID-19 (grupo caso) apresentaram pior qualidade de vida em comparação com pessoas que tiveram COVID-19 e que não desenvolveram complicações respiratórias (grupo controle).

Os achados reforçam a necessidade de estratégias de acompanhamento multiprofissional e do protagonismo da enfermagem na identificação precoce e manejo das sequelas respiratórias da síndrome pós-COVID-19, visando à melhoria da qualidade de vida das pessoas que desenvolveram essa enfermidade.

Para tanto, sugere-se o treinamento da equipe de enfermagem para atuar de forma perspicaz na identificação de alterações na qualidade de vida dessas pessoas acometidas pela COVID-19 e na providência de adaptações necessárias, dentro do seu campo de atuação, para mitigar os efeitos dessas complicações, visto que a enfermagem atua na prevenção de doenças, promoção da saúde, conforto e bem-estar da população.

## Data Availability

Os dados de pesquisa estão disponíveis no corpo do artigo.
